# Is a Higher Protein-Lower Glycemic Index Diet More Nutritious Than a Conventional Diet? A PREVIEW Sub-study

**DOI:** 10.3389/fnut.2020.603801

**Published:** 2020-12-07

**Authors:** Alice Meroni, Roslyn P. Muirhead, Fiona S. Atkinson, Mikael Fogelholm, Anne Raben, Jennie C. Brand-Miller

**Affiliations:** ^1^School of Life and Environmental Sciences and Charles Perkins Centre, The University of Sydney, Sydney, NSW, Australia; ^2^Department of Food and Environmental Sciences, University of Helsinki, Helsinki, Finland; ^3^Department of Nutrition, Exercise, and Sports, Faculty of Science, University of Copenhagen, Copenhagen, Denmark

**Keywords:** zinc, niacin, selenium, vitamin B12, dietary fiber, dietary cholesterol, pre-diabetes

## Abstract

High protein diets and low glycemic index (GI) diets have been associated with improved diet quality. We compared the changes in nutrient intakes of individuals at high risk of developing type-2 diabetes over 3 y who followed either a higher protein-lower GI diet (HPLG) or a conventional moderate protein-moderate GI diet (MPMG). This *post hoc* analysis included 161 participants with overweight and pre-diabetes from the Australian cohort of the PREVIEW study (clinical trial registered in https://www.clinicaltrials.gov/ct2/show/NCT01777893?term=NCT01777893&draw=2&rank=1) who were randomly assigned to a HPLG diet (25% energy from protein, dietary GI ≤ 50, *n* = 85) or a MPMG diet (15% energy from protein, dietary GI ≥ 56, *n* = 76). Food records were collected at 0-mo (baseline) and at 6-, 12-, 24-, and 36-mo (dietary intervention period). Linear mixed models were used to compare the differences in total energy, macro- and micronutrients, dietary GI, glycemic load (GL) and body weight between the two diet groups at the 4 dietary intervention time points. At 3 y, 74% participants from the HPLG diet and 74% participants from the MPMG diet completed the trial. The HPLG group showed significantly higher protein intake and lower dietary GI and GL than the MPMG group (group fixed effect *P* < 0.001 for all three parameters). By 6-, 12-, 24-, and 36-mo there was a 3.0, 2.7, 2.2, and 1.4% point difference in protein intake and 6.2, 4.1, 4.8, and 3.9 GI unit difference between the groups. The intake of energy and saturated fat decreased (mostly in the first 6-mo), while the intake of dietary fiber increased (from mo-0 to mo-12 only) in both diets, with no significant differences between the diets. The dietary intakes of zinc (group fixed effect *P* = 0.05), selenium (*P* = 0.01), niacin (*P* = 0.01), vitamin B12 (*P* = 0.01) and dietary cholesterol (group by time fixed effect *P* = 0.001) were higher in the HPLG group than in the MPMG group. Despite both diets being designed to be nutritionally complete, a HPLG diet was found to be more nutritious in relation to some micronutrients, but not cholesterol, than a MPMG diet.

## Introduction

Weight gain resulting in overweight (BMI ≥ 25–30 kg/m^2^) and obesity (BMI > 30 kg/m^2^) is a major contributor to the risk of developing type 2 diabetes (T2D) (1). Sedentary behaviors and excessive dietary energy intake cause positive energy balance, which is the physiological mechanism behind weight gain (2). Therefore, in order to reduce the risk of chronic complications of obesity, it is critical for individuals with overweight to, firstly, decrease dietary energy intake to induce weight loss and, secondly, to maintain weight loss over time (3, 4).

Modifications in the quality of the habitual diet may also play a major role in the prevention of T2D and diabetes risk factors (5, 6). Diet quality is often defined as the ability to meet recommended nutrient intakes without excessive total energy consumption (7). Interestingly, Shay et al. showed that individuals with overweight and obese, who are at increased risk of developing T2D, had higher total energy intakes but consumed less nutritious foods compared to leaner people (8).

The current study was a secondary analysis of the Australian cohort from the PREVIEW (Prevention of diabetes through lifestyle intervention and population studies in Europe and around the World) intervention study, a large multinational study designed to assess the most effective lifestyle (diet and physical activity) strategy to prevent T2D in high-risk individuals (9). Based on the results from the DiOGenes (Diet, Obesity and Genes) study (10), which showed improved weight loss maintenance over 4-mo by following a HPLG diet, the PREVIEW trial hypothesized that a HPLG dietary intervention would be more effective than a conventional MPMG intervention in preventing T2D (9).

Both diets with a low dietary GI and/or low glycemic load (GL), and diets with higher percentage of total energy from protein have been previously found to be protective against T2D (6, 11). Additionally, low dietary GI and GL showed association with improved adherence to daily recommended nutrient intakes required for good health (12–14). This is likely due to some low GI foods (e.g., fruits, legumes, intact whole grains and dairy products) being particularly rich in micronutrients (13). Similarly, protein-dense foods have a high ratio of micronutrients to energy (15). Therefore, consuming a higher percentage of energy from protein-dense foods and lower dietary GI may enhance diet quality by optimizing nutrient adequacy without increasing the total energy intake (15). Improved diet quality may be a potential mechanism underlying the link between low dietary GI, higher protein intake and T2D prevention.

In the present study, we investigated the changes in nutrient intakes in a group of Australian individuals with overweight/obesity prescribed either a healthy HPLG diet or a healthy MPMG diet over a 3-y period. We hypothesized that a healthy HPLG dietary intervention would be associated with more positive changes in micronutrient density than a healthy MPMG dietary intervention.

## Subjects and Methods

This study was an analysis of the Australian data of the PREVIEW study, a 36-mo, multicenter, randomized clinical intervention trial. Fogelholm et al. have described the PREVIEW study protocol in full (9). Briefly, the PREVIEW study aimed to determine the effects of a healthy HPLG diet [protein 25% of energy intake (en%), carbohydrate (CHO) 45 en%, GI ≤ 50] and of a healthy MPMG diet (protein 15 en%, CHO 55 en%, GI ≥ 56) in combination with two exercise regimens (high-intensity or moderate-intensity exercise) on T2D prevention in males and females aged 25–70 y, with overweight (BMI ≥ 25 kg/m^2^) and pre-diabetes (diagnosis based on the American Diabetes Association criteria) (16). The study comprised of a weight loss phase followed by a weight maintenance phase. During the 8-wk weight loss phase, the participants undertook a low energy diet (LED, 800 kcal/d) with the aim to lose ≥8% of 0-mo body weight to be eligible for the 34-mo weight maintenance phase.

The study participants attended 17 group sessions (8–20 individuals per group) delivered by research dietitians throughout both the weight loss phase and weight maintenance phase. With the support of a behavior modification tool, PREMIT (17), the group sessions provided information on how to adopt one of the two healthy intervention diets. In addition to the group counseling visits, dietary material with instructions on how to achieve the prescribed macronutrient composition and dietary GI, and recipes for each diet were provided to participants to encourage compliance.

In the current study, only individuals from The University of Sydney cohort of PREVIEW were included. Participants were compared based solely on their assigned intervention diet, providing two separate groups for the analyses. Data were collected between August 2013 and March 2018. Measurements from 0-, 6-, 12-, 24-, and 36-mo were used for the analyses of the nutritional assessments. Because the weight maintenance phase commenced after an 8-wk weight loss period, the data assessed at 6-, 12-, 24-, and 36-mo correspond to 4-, 10-, 22-, and 34-mo following one of the two dietary interventions.

All study participants provided written informed consent prior to commencing screening measurements. The study protocol (No X14-0408) was reviewed and approved by the Sydney Local Health District Human Research Ethics Committee—Royal Prince Alfred Hospital (Sydney, Australia) and is in accordance with the Declaration of Helsinki of 1975 as revised in 2008.

Reported intakes of energy, macro- and micronutrients and dietary GI and GL values were assessed at 0-, 6-, 12-, 24-, and 36-mo using 4-d (3 weekdays and 1 weekend day) food diaries. Food records were reviewed by research dietitians during the study to assess the adequacy of the information. Research dietitians entered the individual food records in FoodWorks Professional version 8 (Xyris Software, Brisbane, QLD, Australia, 2015) which contained the Australian food composition data (AUSNUT 2011–2013, AUSFOODS 2015 and AUSBRANDS 2015) and GI values (glucose=100 scale). Missing GI values were obtained from the University of Sydney's online database (http://www.glycemicindex.com). Overall dietary GI and GL were calculated as previously described by Louie et al. (18). Marine-sourced long-chain n-3 polyunsaturated fatty acids (LC n-3 PUFAs) daily reported intake was calculated as the sum of eicosapentaenoic acid (EPA) and docosahexaenoic acid (DHA) daily reported intakes obtained from the FoodWorks Professional analysis.

Energy, macro- and micronutrients and GI and GL values used for the analyses were the mean of the 4-d food diaries. Body weight and height were measured as described in the PREVIEW study protocol (9). Body fat percentage (BF%) and fat free mass percentage (FFM%) were obtained using dual X-ray absorptiometry (DEXA) machine (Discovery W model, Hologic Inc, Bedford, MA, USA).

Nitrogen excretion was measured from 24-h urine collections and was used as a marker of protein intake. Total volume (mL) and weight (g) were measured within a few hours of delivery, then samples were frozen at −80°C in 0.8 mL aliquots (3 tubes). The urea content was analyzed on the Beckman Coulter AU480 Clinical Chemistry autoanalyzer (Beckman Coulter Inc, Brea, California, USA) in 100 μL urine samples in duplicate (average coefficient of variation 1.6%).

## Statistical Analysis

All data are expressed as means (± SD) for 0-mo characteristics, or adjusted means (95% CI). Intention-to-treat analyses were calculated by including all available observations (0- to 36-mo) from study participants who completed a 4-d food diary at 0-mo. Data were analyzed under the assumption that missing data were missing at random. Analyses of nutritional assessments and weight loss were conducted by using analysis of covariance (ANCOVA) linear mixed models. In the model, the dependent variables were the dietary intake and body weight values measured at 6-, 12-, 24-, 36-mo. The independent categorical variables (fixed factors) were the diet group (HPLG and MPMG), time (6-, 12-, 24-, and 36-mo), and the interaction between diet group and time. Time was treated as a categorical variable and an unstructured model was applied for covariance matrix between the repeated measurements. The models were adjusted for 0-mo values, age and sex. If the group main effect was significant, the overall differences (a mean of four timepoints) between groups were compared. If both the group main effect and group-by-time interaction were significant, then between group differences at each timepoint were tested. For the purpose of this analysis, the overall differences between timepoints (a mean of 2 groups) were not compared when the main effect of the time showed significance. To show compliance to the assigned dietary interventions, linear mixed models were also used to report the changes from baseline in en% protein, 24-h nitrogen execration and dietary GI at 6-, 12-, 24-, and 36-mo [data represented as adjusted means (SE)]. A sensitivity analysis was conducted by the per-study protocol at 6-mo, which excluded dropout participants and participants who did not complete a 4-d food diary at 6-mo. In this analysis, the differences between the groups were assessed using ANCOVA adjusted for 0-mo values, age and sex. All statistical analyses were performed using IBM SPSS version 24 (IBM Corporation, New York, NY, USA, 2016). Statistical significance was determined by an α level of 0.05.

## Results

**Figure 1:**
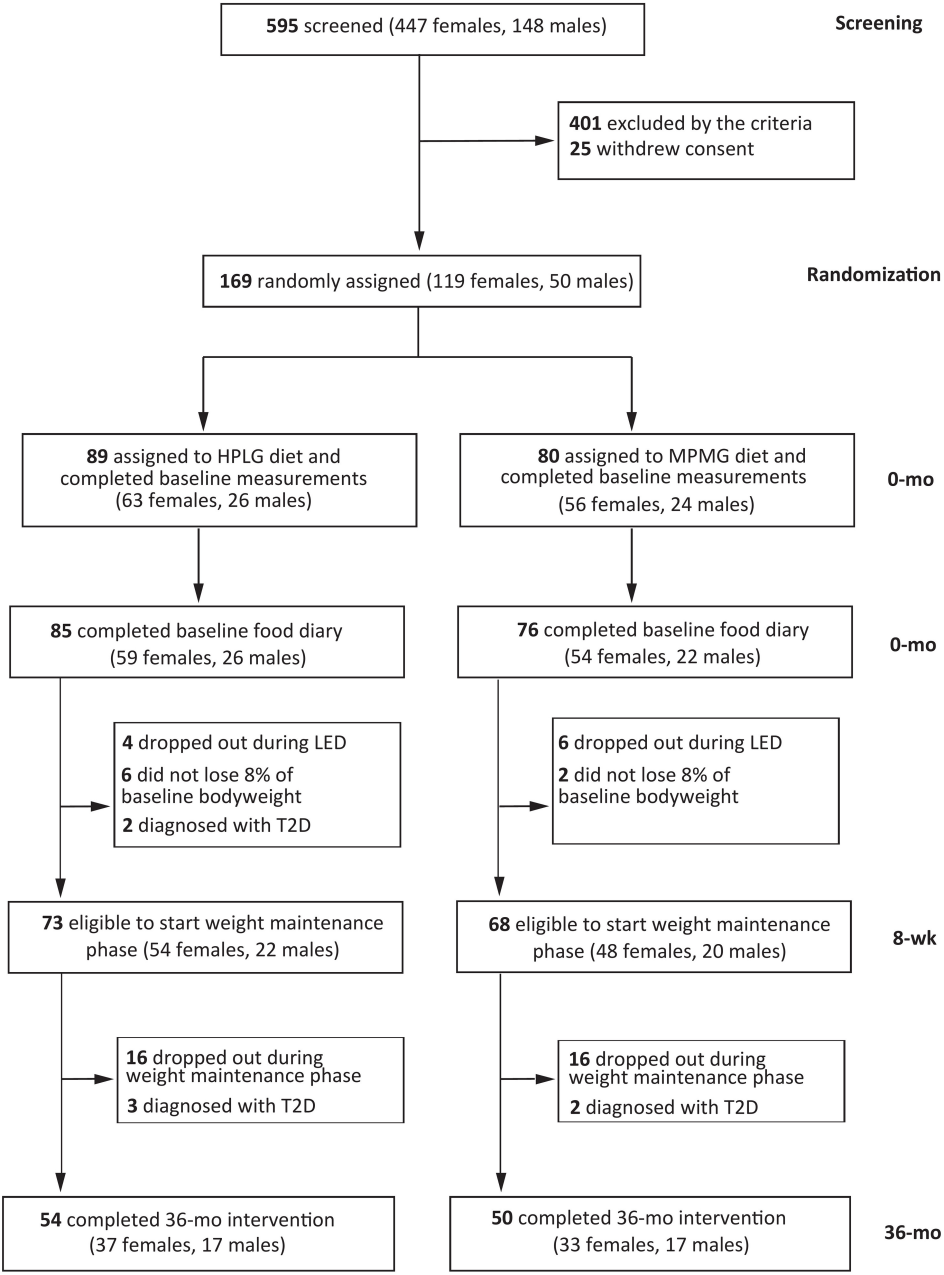
Screening, randomization and follow-up of study participants. Intention-to-treat analysis included all participants who were randomly assigned to the HPLG group or MPMG group and completed the food record during the baseline assessments. HPLG, higher protein-lower glycemic index; MPMG, moderate protein-moderate glycemic index.

Between August 2013 and February 2015, 169 participants were randomly assigned to one of the two dietary interventions (Figure 1). During the 0-mo assessments, 8 participants did not complete the 4-d food diary and were excluded from the intention-to-treat analysis set (Figure 1). Therefore, the analysis of 0-mo data included 161 individuals of whom 85 were assigned to the HPLG diet and 76 to the MPMG diet (Figure 1). During the LED phase, eight participants (six from the HPLG group and two from the MPMG group) did not lose ≥8% body weight, 10 participants (HPLG = 4, MPMG = 6) dropped out of the study and two participants from the HPLG developed T2D. These participants did not continue in the weight maintenance phase (8-wk to 36-mo), leaving 73 participants in the HPLG group and 68 participants in the MPMG group (Figure 1). In total, 104 subjects completed year 3 (74% of those who started the weight maintenance phase), of whom 54 participants were assigned to the HPLG diet (74% of those who started the weight maintenance period) and 50 to the MPMG diet (74%) (Figure 1).

Baseline characteristics of the participants included in the intention-to-treat analysis are summarized in Table 1. In both groups, there were more females than males (69% female in the HPLG group and 71% in the MPMG group), more Caucasians (HPLG 82%, MPMG 83%) than other ethnicities (self-reported) and more individuals in the age group 51–70 y (HPLG 63%, MPMG 62%) than in the age group 25–50 y. The average BMI was 36.3 kg/m^2^ (class II obesity, World Health Organization classification) (19) and the average age was 53 years. Both body fat percentage (BF%) and fat free mass percentage (FFM%) were on average higher in the MPMG (44.4 and 56.2%, respectively) than in the HPLG group (41.7 and 55.6%, respectively). The percentage of energy contributed by protein was 19% protein and daily dietary GI was 54 (moderate, GI Foundation classification) (20) in both groups.

**Table 1:** Baseline characteristics of the intention-to-treat population (*n* = 161)^a^.

	**HPLG group** **(*n* = 85)**	**MPMG group** **(*n* = 76)**
**Ethnicity**		
Caucasian	70 (82%)	63 (83%)
Asian	6 (7%)	5 (7%)
Other	9 (11%)	8 (10%)
**Sex**		
Females	59 (69%)	54 (71%)
Males	26 (31%)	22 (29%)
**Age, years**	52.9 (10.6)	53.0 (10.3)
25–50	31 (37)	29 (38)
51–70	54 (63)	47 (62)
**Screening height, cm**	167.1 (8.3)	167.9 (8.8)
**Weight, kg**	99.1 (18.6)	102.2 (21.7)
**BMI, kg/m**^**2**^	36.3 (7.1)	36.3 (7.2)
**BF, %**	41.7 (11.2)	44.4 (14.4)
**FFM, %**	55.6 (11.0)	56.2 (10.8)
**Energy intake, kJ/d**	9,292 (2,640)	9,165 (2,712)
**Protein, en%**	19.5 (3.5)	19.7 (4.7)
**Fat, en%**	34.3 (5.7)	35.0 (6.3)
**Saturated fat, en%**	11.9 (2.9)	12.6 (3.1)
**Carbohydrate, en%**	40.3 (7.0)	40.2 (7.2)
**Dietary fiber, g**	26.7 (7.8)	26.0 (7.4)
**Dietary GI**	54.1 (5.1)	54.3 (4.5)
**Dietary GL**	119.1 (42.3)	118.4 (45.0)
**Alcohol, en%**	3.5 (4.8)	

a *Values are expressed as n (%) for ethnicity, sex and age and as mean (SD) for all other measurements. BF, body fat; FFM, fat free mass; HPLG, higher protein-lower glycemic index; MPMG, moderate protein-moderate glycemic index*.

**Figure 2:**
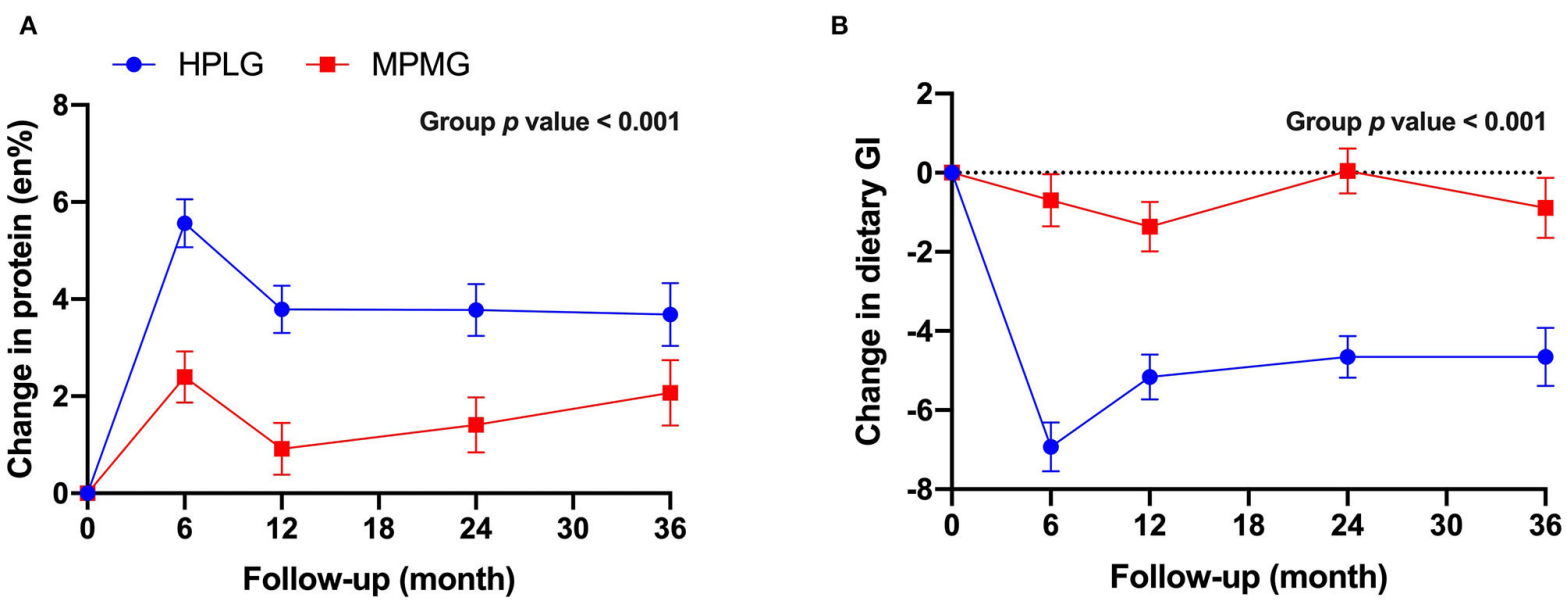
Changes from 0-mo at 6-, 12-, 24-, and 36-mo in protein reported intake **(A)** and dietary glycemic index **(B)**. Values are expressed as mean (SE). HPLG, higher protein-lower glycemic index; MPMG, moderate protein-moderate glycemic index.

Both the HPLG group and the MPMG showed an overall increase from 0-mo (mean of 4 timepoints) in en% protein (Figure 2A). The main effect of the group was significant (*P* < 0.001) indicating that the HPLG group consumed a significantly higher percentage of energy from protein than the MPMG group throughout the intervention. The HPLG group met the 25 en% of protein target only at the 6-mo timepoint and the MPMG group exceeded the 15 en% of protein target at all timepoints (Table 2). Dietary GI showed an overall decrease from 0-mo (mean of four timepoints) in both groups but it was significantly lower in the HPLG group than the MPMG group throughout the intervention (group fixed effect *P* < 0.001) (Figure 2B).

**Table 2:** Body weight, intakes of nutrients, dietary GI and dietary GL at 0-mo and at 6-, 12-, 24-, and 36-mo after the initiation of the dietary intervention in the HPLG and MPMG groups (intention-to-treat analysis population, *n* = 161)^a^.

**Measure and** **Group**	**0-mo**	**6-mo**	**12-mo**	**24-mo**	**36-mo**	***P*****-value for Fixed Effects^d^**		
						**Group**	**Time**	**Group by** **Time**
**Weight, kg**								
HPLG^b^	99.1 (18.6)	87.1 (85.9, 88.3)	89.4 (87.7, 91.1)	93.0 (91.1, 94.9)	93.7 (92.0, 95.4)	0.91	<0.001	0.63
MPMG^c^	102.2 (21.7)	87.3 (86.0, 88.6)	89.9 (88.1, 91.7)	92.6 (90.5, 94.7)	93.8 (92.0, 95.6)			
**Energy, kJ/d**								
HPLG	9,296 (2,478)	6,872 (6,523, 7,221)	7,230 (6,832, 7,629)	7,274 (6,749, 7,799)	7,127 (6,685, 7,569)	0.84	0.11	0.96
MPMG	9,145 (2,472)	6,921 (6,550, 7,292)	7,167 (6,731, 7,602)	7,143 (6,579, 7,708)	7,078 (6,618, 7,539)			
**Protein, en%**								
HPLG	19.5 (3.5)	25.2 (24.2, 26.2)	23.4 (22.5, 24.2)	23.4 (22.4, 24.5)	23.3 (22.1, 24.6)	<0.001	<0.001	0.48
MPMG	19.7 (4.7)	22.0 (21.0, 23.1)	20.6 (19.5, 21.6)	21.1 (19.9, 22.2)	21.7 (20.4, 23.1)			
**Protein, g/d**								
HPLG	104.8 (30.3)	101.3 (96.6, 105.9)	98.1 (93.1, 103.1)	97.2 (91.9, 102.6)	95.7 (89.7, 101.7)	<0.001	0.28	0.53
MPMG	104.0 (35.9)	88.3 (83.4, 93.2)	85.7 (80.3, 91.2)	86.6 (80.9, 92.3)	89.0 (82.7, 95.3)			
**CHO, en%**								
HPLG	40.3 (7.0)	36.8 (35.4, 38.3)	37.1 (35.8, 38.4)	36.3 (34.6, 37.9)	36.7 (34.8, 38.7)	<0.001	0.08	0.12
MPMG	40.2 (7.2)	42.4 (40.9, 44.0)	41.6 (40.1, 43.1)	40.2 (38.4, 42.0)	38.3 (36.3, 40.3)			
**CHO, g/d**								
HPLG	218.6 (68.6)	147.7 (137.3, 158.1)	156.3 (146.2, 166.4)	151.5 (140.2, 162.8)	153.2 (141.0, 165.5)	0.009	0.21	0.22
MPMG	216.4 (72.4)	173.1 (162.0, 184.3)	174.0 (162.0, 184.3)	167.1 (155.0, 179.3)	157.5 (144.8, 170.2)			
**Fat, en%**								
HPLG	34.3 (5.7)	31.0 (29.5, 32.5)	33.1 (31.6, 34.7)	33.4 (31.8, 35.1)	33.5 (31.7, 35.3)	0.97	<0.001	0.55
MPMG	34.9 (6.3)	30.4 (28.8, 32.0)	32.6 (30.9, 34.3)	33.1 (31.4, 34.9)	34.8 (33.0, 36.7)			
**Saturated fat, en%**								
HPLG	11.9 (2.8)	10.1 (9.4, 10.8)	10.5 (9.8, 11.2)	10.8 (10.1, 11.6)	10.9 (10.0, 11.7)	0.84	0.01	0.55
MPMG	12.6 (3.0)	9.9 (9.2, 10.6)	10.5 (9.7, 11.3)	10.6 (9.8, 11.5)	11.6 (10.7, 12.5)			
**Starch, g/d**								
HPLG	127.0 (45.0)	76.4 (69.3, 83.4)	83.8 (77.1, 90.5)	80.5 (72.9, 88.2)	83.3 (74.8, 91.8)	<0.001	0.27	0.12
MPMG	122.8 (46.7)	100.3 (92.8, 107.8)	100.7 (93.4, 108.1)	97.7 (89.4, 105.9)	90.0 (81.4, 105.9)			
**Sugars, g/d**								
HPLG	89.0 (36.1)	69.4 (63.5, 75.3)	70.8 (65.4, 76.1)	68.7 (63.1, 74.2)	67.8 (60.9, 74.7)	0.82	0.34	0.89
MPMG	91.4 (36.6)	72.3 (66.0, 78.7)	72.4 (66.6, 78.3)	68.3 (62.3, 74.2)	66.6 (59.5, 73.8)			
**Dietary fiber, g/d**								
HPLG	26.7 (7.7)	26.7 (24.9, 28.5)	28.8 (26.9, 30.8)	25.2 (23.3, 26.9)	26.4 (24.8, 28.0)	0.53	<0.001	0.02
MPMG	26.0 (7.4)	27.1 (25.2, 29.0)	27.3 (25.2, 29.5)	26.0 (24.2, 27.8)	24.1 (22.4, 25.9)			
**Dietary GI**								
HPLG	54.1 (5.1)	47.1 (45.9, 48.3)	48.9 (47.8, 50.0)	49.4 (48.3, 50.4)	49.4 (47.9, 50.8)	<0.001	0.04	0.10
MPMG	54.3 (4.5)	53.3 (52.0, 54.7)	52.7 (51.4, 53.9)	54.1 (53.0, 55.2)	53.2 (51.6, 54.7)			
**Dietary GL**								
HPLG	119.1 (42.3)	70.7 (64.7, 76.7)	76.6 (70.8, 82.3)	74.8 (68.6, 81.0)	76.9 (69.5, 84.3)	<0.001	0.49	0.07
MPMG	118.4 (45.0)	92.6 (86.2, 99.0)	91.7 (85.4, 97.9)	90.7 (84.1, 97.4)	83.7 (76.1, 91.3)			
**Cholesterol, mg/d**								
HPLG	381.5 (178.5)	350.2 (322.8, 377.5)^**^	315.9 (286.3, 345.5)^*^	337.3 (296.2, 278.4)^*^	320.2 (282.2, 358.2)^*^	0.001	0.34	0.001
MPMG	363.3 (172.1)	225.8 (196.7, 254.8)^**^	264.9 (232.4, 297.4)^*^	282.1 (238.1, 326.1)^*^	294.8 (254.6, 335.0)^*^			
**MUFAs, g/d**								
HPLG	34.4 (13.0)	22.9 (20.9, 24.9)	26.9 (24.6, 29.2)	26.5 (24.1, 29.0)	26.3 (23.6, 29.1)	0.96	<0.001	0.58
MPMG	33.5 (12.7)	23.0 (20.9, 25.1)	25.5 (23.0, 28.0)	26.5 (23.9, 29.2)	27.9 (25.0, 30.9)			
**PUFAs, g/d**								
HPLG	14.6 (5.4)	11.4 (10.4, 12.5)	12.0 (10.8, 13.2)	11.6 (10.5, 12.7)	11.3 (10.2, 12.3)	0.91	0.04	0.21
MPMG	14.2 (6.1)	10.3 (9.1, 11.4)	12.0 (10.7, 13.4)	12.1 (11.0, 13.3)	11.6 (10.5, 12.8)			
**LC n-3 PUFA, mg/d**								
HPLG	436.9 (491.8)	647.2 (506.9, 787.5)	521.8 (398.2, 645.3)	553.9 (403.0, 704.9)	418.0 (271.3, 564.7)	0.08	0.52	0.11
MPMG	393.1 (490.0)	404.9 (255.5, 554.2)	425.8 (289.7, 561.9)	372.7 (209.9, 535.4)	442.6 (287.6, 597.4)			
**LA, g/d**								
HPLG	11.9 (4.7)	8.6 (7.8, 9.5)	9.4 (8.3, 10.4)	9.2 (8.2, 10.2)	9.0 (8.1, 9.9)	0.53	0.01	0.59
MPMG	11.5 (5.0)	8.3 (7.4, 9.2)	9.8 (8.7, 10.9)	9.9 (8.9, 11.0)	9.3 (8.4, 10.3)			
**ALA, g/d**								
HPLG	1.7 (0.7)	1.6 (1.4, 1.9)	1.7 (1.4, 1.9)	1.5 (1.3, 1.7)	1.5 (1.3, 1.7)	0.07	0.69	0.20
MPMG	1.9 (1.3)	1.2 (1.0, 1.5)	1.4 (1.2, 1.7)	1.4 (1.2, 1.6)	1.4 (1.2, 1.6)			
**Alcohol, en%**								
HPLG	3.5 (4.8)	3.5 (2.6, 4.4)	2.9 (2.1, 3.8)	3.8 (2.5, 5.2)	3.4 (2.3, 4.5)	0.03	0.46	0.91
MPMG	2.9 (5.6)	2.0 (1.0, 2.9)	1.9 (1.0, 2.8)	2.5 (1.1, 4.0)	2.2 (1.1, 3.4)			
**Zinc, mg/d**								
HPLG	12.1 (4.1)	11.7 (10.7, 12.6)	11.7 (10.9, 12.4)	11.0 (10.1, 11.8)	11.2 (10.3, 12.1)	0.05	0.23	0.44
MPMG	12.4 (5.1)	10.9 (10.0, 11.9)	10.3 (9.4, 11.1)	10.0 (9.1, 10.9)	10.8 (9.9, 11.8)			
**Selenium**, **μg/d**								
HPLG	108.1 (42.7)	107.8 (94.5, 121.1)	92.4 (84.4, 100.3)	96.2 (88.8, 103.6)	99.5 (85.7, 113.3)	0.01	0.53	0.34
MPMG	100.9 (38.0)	88.0 (73.8, 102.1)	88.1 (79.5, 96.7)	85.5 (77.3, 93.6)	81.6 (66.9, 96.2)			
**Niacin (NE), mg/d**								
HPLG	26.4 (9.8)	25.9 (23.7, 28.1)	24.5 (22.7, 26.4)	24.2 (22.4, 25.9)	25.4 (23.0, 27.7)	0.01	0.38	0.72
MPMG	26.0 (10.5)	23.0 (20.6, 25.3)	21.9 (19.9, 23.9)	22.1 (20.2, 24.0)	21.2 (18.8, 23.7)			
**Vitamin B12**, **μg/d**								
HPLG	5.3 (2.7)	5.6 (5.1, 6.1)	5.2 (4.7, 5.6)	5.0 (4.5, 5.5)	5.1 (4.4, 5.8)	0.01	0.17	0.71
MPMG	5.4 (3.7)	4.6 (4.1, 5.2)	4.4 (3.9, 4.8)	4.4 (3.8, 4.9)	4.5 (3.8, 5.3)			

a Values are expressed as the mean (SD) or adjusted mean (95% CI). The adjusted means with a double asterisk or single asterisk indicate a statistically significant difference between the groups at the timepoint (^**^P < 0.001; ^*^P < 0.05). GI, glycemic index; GL, glycemic load; HPLG, higher protein-lower glycemic index diet; MPMG, moderate protein-moderate glycemic index diet; NE, niacin equivalent.

b *Number of participants 85, 68, 64, 60 and 54 at 0-, 6-, 12-, 24-, and 36-mo, respectively*.

c *Number of participants 76, 60, 52, 52 and 50 at 0-, 6-, 12-, 24-, and 36-mo, respectively*.

d *P-values show the significance of the fixed effects for changes in a variable as assessed by linear mixed model adjusted for each measurement 0-mo values, age and sex*.

**Figure 3:**
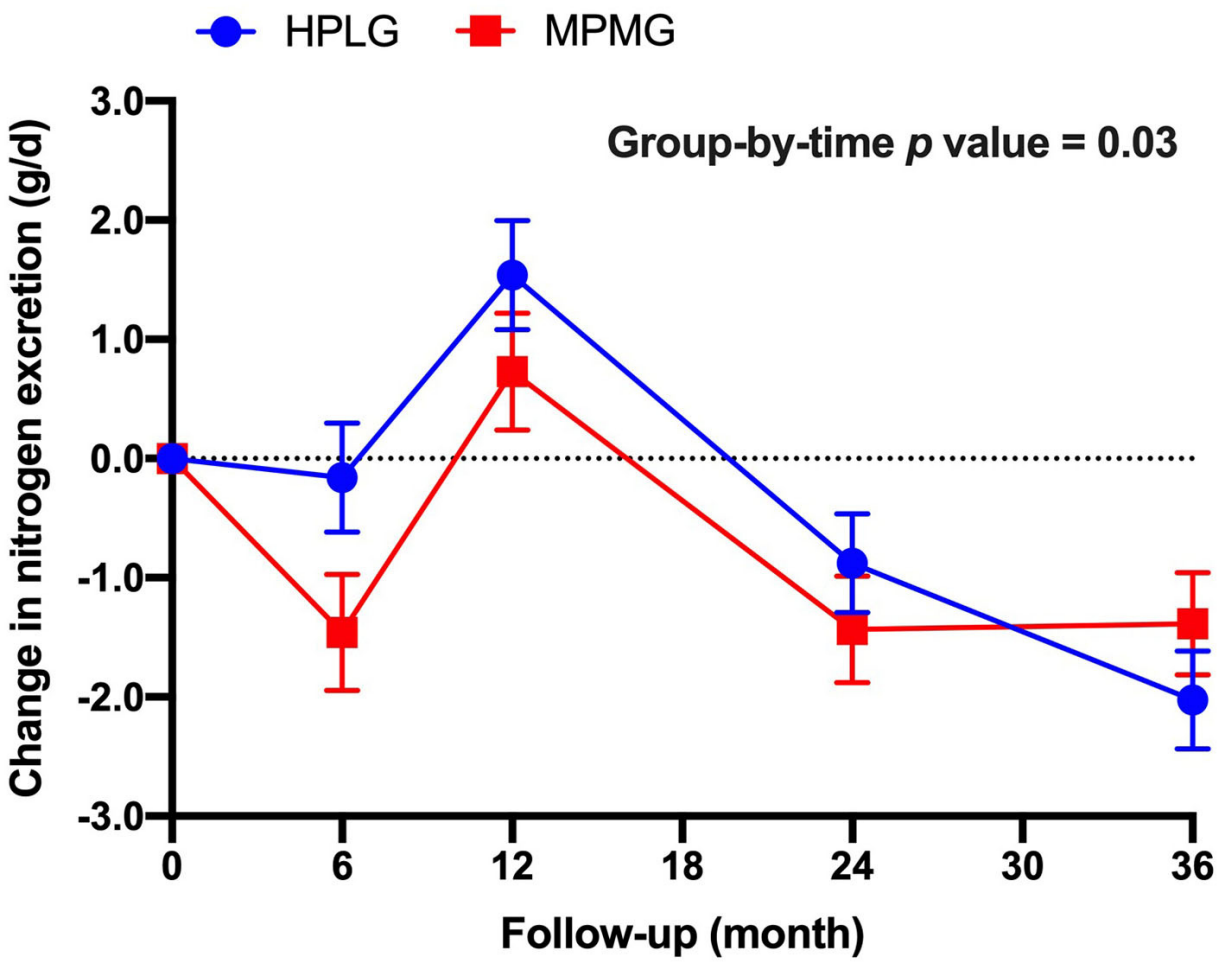
Changes from 0-mo at 6-, 12-, 24-, and 36-mo in nitrogen execration (24 h). Values are expressed as mean (SE). HPLG, higher protein-lower glycemic index; MPMG, moderate protein-moderate glycemic index.

There was a significant group-by-time effect (*P* = 0.03) in 24-h nitrogen excretion (a marker of protein intake), but the main effect of the group did not reach statistical significance (*P* = 0.16) (Figure 3). Nitrogen excretion (24-h), was higher in the HPLG group than in the MPMG group at 6-, 12-, and 24-mo, but higher in the MPMG than in the HPLG at 36-mo.

Table 2 shows body weight and the reported intake of macronutrients, dietary GI, dietary GL, Zinc, Selenium, Niacin and Vitamin B12 at 0-, 6-, 12-, 24-, and 36-mo in the intention-to-treat population. Body weight and energy intake decreased in both the HPLG and MPLG diet groups (with the greatest difference between the groups at 6-mo) and there were no significant differences between the groups during the intervention (group fixed effect *P* = 0.91 and *P* = 0.84, respectively). Fixed effects of the group were significant for the changes in the groups' absolute intake (g/d) of protein (*P* < 0.001), en% carbohydrate (*P* < 0.001), absolute intake (g/d) of carbohydrate (*P* = 0.009), starch (*P* < 0.001) and dietary GL (*P* < 0.001) (Table 2). There was a significant group-by-time effect (*P* = 0.02) but not a significant group effect (*P* = 0.52) for dietary fiber, with both diets showing an increase in intake from 0- to 12-mo. At 12-mo, the increase in dietary fiber from 0-mo was greater in the HPLG group than in the MPMG group but the difference in intake was not significant between the diets.

The percentage of energy contributed by total fat and by saturated fat showed a decrease in both groups at 6-mo, but the decrease was attenuated at 12-, 24-, and 36-mo. The main effect of the group for the en% total fat and en% saturated fat did not reach statistical significance (*P* = 0.97 and *P* = 0.84, respectively). At 6-mo, there was a significant difference (*P* < 0.001) in groups' cholesterol intake [adjusted means: HPLG 350.2 mg (95% CI: 322.8, 377.5), MPMG 225.8 mg (196.7, 254.8)]. The differences remained statistically significant (*P* < 0.05), although they were less pronounced at 12-, 24-, and 36-mo. The fixed effect of the group was significant for en% alcohol (*P* = 0.03) but not the group-by-time interactions, therefore the effect of the group did not differ between timepoints (Table 2).

Zinc, selenium, niacin and vitamin B12 showed significant group effects (*P* = 0.05 for zinc and *P* = 0.01 for the other three micronutrients), with the HPLG group overall reportedly consuming higher intakes of these micronutrients than the MPMG group. However, the group fixed effects were not significant suggesting that the effect of the group did not differ between timepoints.

Supplementary Tables 1, 2 show the micronutrient (minerals and vitamins, excluding zinc, selenium, niacin and vitamin B12) reported intakes at 0-, 6-, 12-, 24-, and 36-mo. Calcium, iron, potassium, sodium, phosphorus, magnesium, iodine, thiamine, riboflavin, vitamin B6, dietary folate equivalent (DFE), vitamin A, vitamin C and vitamin E did not show any significant group-by-time effects nor group effects.

Compared to the intention-to-treat analysis, in the per-study protocol analysis, the HPLG group showed a greater consumption of α-linolenic acid (ALA) and of LC n-3 PUFA at 6-mo than the MPMG group (Supplementary Table 3). However, significant differences were no longer found between the groups' reported intakes of zinc and niacin (Supplementary Table 3).

## Discussion

To the best of our knowledge, this is the first study comparing the nutrient density of a healthy HPLG diet and of a healthy MPMG diet prescribed for a 3-y period. We found that, with comparable reductions in energy intake between the two groups during the intervention, individuals following the HPLG diet consumed higher intakes of some micronutrients than those assigned to the MPMG diet. Specifically, the HPLG group had higher intakes of zinc, selenium, niacin and vitamin B12 compared to the MPMG group. These results support our hypothesis that a HPLG diet is more micronutrient-dense than a conventional diet. Conversely, participants in the MPMG group showed lower intake of dietary cholesterol than those prescribed to the HPLG diet.

Previous studies have reported that higher protein diets and lower GI/GL diets improved diet quality and protection against chronic conditions (13, 15, 21, 22). Zinc, selenium, niacin and vitamin B12, for which reported intake was higher in the HPLG group compared to the MPMG group, are predominantly found in animal sources of protein. Some studies have linked high protein, particularly animal protein to increased risk of chronic diseases, especially when the protein sources are of poor quality (23). Since the en% contributed by total fat and saturated fat decreased during the intervention, it suggests that the HPLG intervention achieved the prescribed 25 en% from protein by favoring the consumption of lean meats (e.g., skinless poultry and fish) to meats higher in saturated fat, as recommended. This was also supported by the tendency to increased reported intake of dietary LC n-3 PUFA (predominantly found in oily fish) in the HPLG group compared to the MPMG group until the 2-y analysis. The consumption of lean animal sources of proteins has been previously linked with increased nutrients of particular concern, including vitamin B12, zinc, and selenium (15). In the PREVIEW study Australia, the majority of participants (63 and 62% in the HPLG and MPMG group, respectively) were aged 51–70 y, consistent with the higher prevalence of pre-diabetes in the older population. Older adults have increased risk of vitamin B12 deficiency due to the reduced physiological ability to absorb vitamin B12 with aging (24). Moreover, poor zinc status has been linked with decreased insulin secretion and insulin insensitivity (25) and low blood selenium concentrations with an increased incidence of cardiovascular disease (CVD) (26). Therefore, in the long-term, individuals with pre-diabetes may particularly benefit from a HPLG diet which is more likely to provide adequate intakes of vitamin B12 [Estimated Average Requirements (EAR) for Australia and New Zealand = 2.0 μg/day for both males and females] (27), zinc (EAR = 12 and 6.5 mg/day for males and females, respectively) (28) and selenium (EAR = 60 and 50 μg/day for males and females, respectively) (29).

Dietary cholesterol, which is abundant in protein-dense foods, such as eggs, and low GI foods such as dairy products, was not surprisingly higher in the HPLG intervention. Despite some evidence suggesting that dietary cholesterol increases the risk of CVD (30, 31), the link between dietary cholesterol and cardiometabolic health remains controversial (32). Furthermore, any potential cardiovascular risk derived from higher dietary cholesterol in the HPLG diet may have been attenuated by the greater increase in dietary fiber observed at 12-mo and 36-mo in the HPLG group compared to the MPMG group and by the reduction in en% contributed by saturated fat in both dietary groups. Indeed, dietary fiber has been proposed to inhibit the absorption of cholesterol and improve control of cholesterol concentration (33) and a lower intake of saturated fat has shown to be cardioprotective for people with pre-diabetes, a population at higher risk of cardiovascular complications (34).

While the differences in dietary protein, carbohydrate, GI and GL between the two groups were statistically significant, by 3 y, the participants were not achieving the targeted separation of 10% points for protein and carbohydrate reported intake and of 6 GI units between the two interventions. This is related to the MPMG group consuming more protein (5 percentage points higher intake) and less dietary GI (2 GI units) than the targeted 15 en% from protein and dietary GI ≥ 56. In both groups, the baseline values of protein (~20 en%) were higher and the baseline values of carbohydrate (~40 en%) were lower than the averages observed in the 2011–2012 Australian National Nutrition and Physical Activity Survey (protein 18–19 en% and carbohydrate 42–45 en%) (35). This pattern was consistent with that of a moderately high protein-lower carbohydrate diet. Therefore, more effort was required by individuals in the MPMG group to make changes to their habitual diet, which may have accounted for the failure of the group to comply with the prescribed dietary targets. Furthermore, the GI concept is well-known in Australia due to efforts of non-governmental organizations to educate the population about GI (20). “Low-GI” labeled foods are also widely available in supermarkets (36). For these reasons, individuals in the MPMG group may have been less willing to choose higher GI food options to increase their dietary GI. These findings should be considered in the planning of future studies.

Surprisingly, the en% from alcohol reported intake decreased significantly more in the MPMG group throughout the weight maintenance phase, despite both groups receiving the same advice regarding limiting alcohol consumption. However, at 3 y, alcohol intake was still below 5% of dietary energy intake in both groups as recommended by the National Health and Medical Research Council of Australia to avoid weight gain, nutritional inadequacy and harm from alcohol consumption (37).

A notable strength of our study was the use of a behavior modification tool specifically developed for the PREVIEW trial (PREMIT, the PREVIEW behavior Modification Intervention Toolbox) (17). Because the two intervention diets were designed to be healthy, it is plausible that the behavior modification tool by itself was effective in imparting more positive nutrition habits in both groups. This is evidenced by the overall reduced energy intake, maintained weight loss and nutritional adequacy achieved by the entire cohort.

Food diary entries were reviewed by research dietitians with the study participants. This added strength to our study because it improved completeness of the food diaries and ensured that evident mistakes were corrected before the data were entered into the nutritional database. Other major strengths include the randomized controlled intention-to-treat design, which minimized the potential of selection bias, and the long dietary intervention period (36-mo). There were similar dropouts in both diet arms. Indeed, any modification in the habitual diet is considered successful when implemented in the long-term.

This study also has some limitations. The MPMG group did not meet the protein, carbohydrate and dietary GI targets of the diet. Higher dietary adherence may have enabled stronger conclusions to be made. Despite 4-d food diaries being reasonably accurate, self-reported methods to measure nutritional intake are prone to under- and over-reporting and any food composition database is limited by inherent variation (38). However, in this study, measurements of urinary nitrogen excretion (higher in the HPLG group at 6-, 12-, and 24-mo) confirmed the differences in protein reported intake between the interventions when the compliance to the assigned diets was reported higher in 4-d food diaries (a strength). Finally, it could be argued that an increase in protein from 20 to 25 en% in the HPLG diet may/may not be clinically relevant to whole body amino acid and nitrogen maintenance.

In conclusion, in the Australian context, long-term adherence to a HPLG diet—denser in zinc, selenium, niacin and vitamin B12—may offer greater nutritional benefit than a conventional MPMG diet to individuals with pre-diabetes and may be linked with chronic disease prevention. Future research is needed to investigate the effects of improved diet quality through higher protein intake and lower dietary GI on biomarkers of optimum health. The environmental impacts of consuming diets with varying amounts and sources of protein must also be considered (39).

## Data Availability Statement

The original contributions presented in the study are included in the article/Supplementary Materials, further inquiries can be directed to the corresponding author/s.

## Ethics Statement

The studies involving human participants were reviewed and approved by Sydney Local Health District Human Research Ethics Committee – Royal Prince Alfred Hospital (Sydney, Australia). The patients/participants provided their written informed consent to participate in this study.

## Author Contributions

AM, RM, FA, and JB-M: designed this research. AR and MF: designed the protocol for the overall PREVIEW intervention study. AM and RM: conducted the research and analyzed the data. AM: performed the statistical analysis, wrote the manuscript, and has primary responsibility for the final content. All authors contributed to the article and approved the submitted version.

## Conflict of Interest

FA and JB-M manage The University of Sydney's glycemic index testing service and are directors of the Glycemic Index Foundation, a not-for-profit health promotion charity. JB-M is an author of books in The New Glucose Revolution series (De Capo, Cambridge, MA). The remaining authors declare that the research was conducted in the absence of any commercial or financial relationships that could be construed as a potential conflict of interest.
