# Supplementary material for: Is a Higher Protein-Lower Glycemic Index Diet More Nutritious Than a Conventional Diet? A PREVIEW Sub-study
Source: Front Nutr. 2020 Dec 7;7:603801. doi: 10.3389/fnut.2020.603801 (PMC7750310; doi:10.3389/fnut.2020.603801)
Supplement: Supplementary file 1 [file Table_1.pdf]

SUPPLEMENTAL TABLE 1 Intakes of minerals at 0-mo and at 6-, 12- , 24- and 36-mo after the initiation of the dietary intervention in the HPLG and MPMG groups (intention-to-treat analysis population, n= 161)[1]

Measure and group	0-mo	6-mo	12-mo	24-mo	36-mo	*P*-value for Fixed Effects[4]		
Calcium, mg/d						Group	Time	Group by Time
HPLG[2]	912.3 (281.2)	942.1 (865.2, 1019.0)	941.0 (864.0, 1018.0)	866.7 (799.1, 934.3)	897.0 (807.3, 986.7)	0.31	0.001	0.73
MPMG[3]	948.1 (318.3)	1026.3 (944.5, 1108.1)	981.3 (897.1, 1065.5)	889.3 (816.7, 961.9)	926.6 (831.7, 1021.5)			
Iron, mg/d								
HPLG	12.5 (3.8)	11.1 (10.4, 11.8)	11.4 (10.7, 12.1)	10.7 (9.9, 11.4)	10.8 (10.0, 11.6)	0.65	0.25	0.86
MPMG	11.9 (3.5)	11.1 (10.4, 11.8)	11.0 (10.2, 11.8)	10.7 (9.9, 11.4)	10.6 (9.7, 11.4)			
Potassium, mg/d								
HPLG	3342 (892)	3430 (3250, 3610)	3436 (3249, 3624)	3206 (3034, 3377)	3301 (3092, 3509)	0.15	0.00	0.42
MPMG	3307 (918)	3362 (3170, 3553)	3213 (3007, 3418)	3118 (2933, 3302)	3051 (2831, 3272)			
Sodium, mg/d								
HPLG	2897 (1070)	2142 (1944, 2340)	2194 (2002, 2387)	2183 (1999, 2367)	2143 (1895, 2391)	0.74	0.70	0.75
MPMG	2813 (1109)	2115 (1904, 2325)	2260 (2048, 2473)	2160 (1963, 2358)	2262 (2003, 2520)			
Phosphorus, mg/d								
HPLG	1656 (459)	1620 (1541, 1699)	1611 (1521, 1701)	1528 (1451, 1605)	1557 (1463, 1651)	0.15	0.01	0.91
MPMG	1673 (485)	1558 (1474, 1642)	1524 (1425, 1623)	1485 (1402, 1568)	1472 (1372, 1572)			
Magnesium, mg/d								
HPLG	382.6 (105.6)	424.9 (393.2, 456.6)	404.2 (376.8, 431.6)	371.4 (348.7, 349.1)	386.4 (361.1, 411.8)	0.15	<0.001	0.59
MPMG	395.7 (115.7)	405.1 (371.4, 438.9)	391.9 (361.8, 422.0)	362.0 (337.6, 386.4)	347.7 (321.0, 374.4)			
Iodine, μg/d								
HPLG	196.4 (64.5)	164.4 (151.4, 177.4)	161.7 (149.6, 173.7)	158.5 (144.6, 172.4)	153.1 (136.7, 169.5)	0.14	0.71	0.92
MPMG	185.4 (58.7)	170.7 (157.0, 184.5)	171.0 (157.4, 184.5)	171.9 (156.9, 187.0)	167.2 (150.0, 184.4)			

[1]Values are expressed as the mean (SD) or adjusted mean (95% CI). GI, glycemic index; GL, glycemic load; HPLG, higher protein-lower glycemic index diet; MPMG, moderate protein-moderate glycemic index diet.
[2]Number of participants 85, 68, 64, 60 and 54 at 0-, 6-, 12-, 24- and 36-mo, respectively.
[3]Number of participants 76, 60, 52, 52 and 50 at 0-, 6-, 12-, 24- and 36-mo, respectively.
[4]*P* values show the significance of the fixed effects for changes in a variable as assessed by linear mixed model adjusted for each measurement 0-mo values, age and sex.

SUPPLEMENTAL TABLE 2 Intakes of vitamins at 0-mo and at 6-, 12- , 24- and 36-mo after the initiation of the dietary intervention in the HPLG and MPMG groups (intention-to-treat analysis population, n= 161)[1]

[1]Values are expressed as the mean (SD) or adjusted mean (95% CI). GI, glycemic index; GL, glycemic load; HPLG, higher protein-lower glycemic index diet; MPMG, moderate protein-moderate

Measure and group	0-mo	6-mo	12-mo	24-mo	36-mo	*P*-value for Fixed Effects[4]		
Thiamine, mg/d						Group	Time	Group by Time
HPLG[2]	1.8 (0.8)	1.3 (1.2, 1.5)	1.5 (1.3, 1.7)	1.3 (1.1, 1.5)	1.5 (1.3, 1.7)	0.12	0.25	0.27
MPMG[3]	1.6 (0.7)	1.5 (1.3, 1.6)	1.6 (1.4, 1.8)	1.7 (1.5, 1.9)	1.5 (1.3, 1.8)			
Riboflavin, mg/d								
HPLG	2.1 (0.7)	2.1 (1.9, 2.2)	2.1 (1.9, 2.3)	1.9 (1.7, 2.0)	2.0 (1.8, 2.2)	0.94	0.01	0.80
MPMG	2.1 (0.8)	2.1 (1.9, 2.2)	2.1 (1.9, 2.3)	1.9 (1.8, 2.1)	2.0 (1.7, 2.2)			
Vitamin B6, mg								
HPLG	1.7 (0.8)	1.5 (1.4, 1.7)	1.6 (1.4, 1.7)	1.5 (1.4, 1.6)	1.6 (1.4, 1.8)	0.19	0.47	0.89
MPMG	1.6 (0.6)	1.5 (1.3, 1.6)	1.5 (1.4, 1.6)	1.4 (1.3, 1.6)	1.5 (1.3, 1.6)			
DFE, µg/d								
HPLG	345.3 (106.4)	357.6 (330.6, 384.5)	341.3 (314.7, 367.8)	338.5 (313.0, 364.1)	326.8 (298.2, 355.4)	0.79	0.14	0.98
MPMG	349.4 (117.2)	359.3 (330.6, 387.9)	344.7 (315.5, 373.9)	340.0 (312.5, 367.5)	336.2 (305.8, 366.5)			
Vitamin A (RE), µg/d								
HPLG	1027 (522)	1087 (954, 1221)	1122 (949, 1295)	1102 (961, 1244)	1251 (1029, 1402)	0.21	0.78	0.80
MPMG	1088 (1019)	1030 (888, 1172)	1031 (841, 1220)	1009 (856, 1162)	1019 (821, 1216)			
Vitamin C, mg/d								
HPLG	104.2 (59.1)	126.0 (109.5, 142.6)	113.9 (97.0, 130.7)	114.5 (100.3, 128.7)	124.1 (109.8, 138.5)	0.69	0.34	0.37
MPMG	97.6 (45.8)	128.6 (111.0, 146.2)	129.8 (111.2, 148.3)	115.8 (100.6, 131.1)	117.4 (102.2, 132.6)			
Vitamin E, mg/d								
HPLG	13.9 (4.8)	11.4 (10.3, 12.5)	12.9 (11.6, 14.2)	14.0 (11.4, 16.6)	12.6 (11.2, 14.0)	0.43	0.02	0.75
MPMG	13.2 (4.6)	11.1 (9.9, 12.2)	12.5 (11.1, 13.9)	12.1 (9.7, 14.9)	12.7 (11.2, 14.2)			

glycemic index diet; RE, retinol equivalent.
[2]Number of participants 85, 68, 64, 60 and 54 at 0-, 6-, 12-, 24- and 36-mo, respectively.
[3]Number of participants 76, 60, 52, 52 and 50 at 0-, 6-, 12-, 24- and 36-mo, respectively.
[4]*P* values show the significance of the fixed effects for changes in a variable as assessed by linear mixed model adjusted for each measurement 0-mo values, age and sex.

SUPPLEMENTAL TABLE 3 Intakes of nutrients, dietary GI and dietary GL at 6-mo after the initiation of the dietary intervention in the HPLG and MPMG groups (per-protocol analysis population, n= 128)

Measure	Group	n	6-mo[1]	Between Groups *P*-value[2]
Energy, kJ/d	HPLG	68	7290 (6882, 7697)	0.68
	MPMG	60	7164 (6711, 7616)	
Protein, en%	HPLG	68	25.2 (24.2, 26.2)	<0.001
	MPMG	60	22.0 (20.1, 23.1)	
Protein, g/d	HPLG	68	101.5 (96.9, 106.2)	<0.001
	MPMG	60	88.1 (83.1, 93.1)	
CHO, en%	HPLG	68	36.8 (35.3, 38.2)	<0.001
	MPMG	60	42.4 (40.8., 43.9)	
CHO, g/d	HPLG	68	148.1 (137.6, 158.6)	0.001
	MPMG	60	173.6 (162.5, 184.8)	
Fat, en%	HPLG	68	34.1 (32.8, 35.4)	0.51
	MPMG	60	34.8 (33.3, 36.2)	
Starch, g/d	HPLG	68	76.6 (69.6, 83.6)	<0.001
	MPMG	60	100.3 (92.8, 107.7)	
Sugars, g/d	HPLG	68	69.5 (63.6, 75.4)	0.46
	MPMG	60	72.7 (66.4, 79.0)	
Dietary fiber, g/d	HPLG	68	26.7 (24.9, 28.5)	0.88
	MPMG	60	26.9 (24.9, 28.8)	
Dietary GI	HPLG	68	47.1 (45.9, 48.3)	<0.001
	MPMG	60	53.4 (52.1, 54.8)	
Dietary GL	HPLG	68	71.1(65.1, 77.2)	<0.001
	MPMG	60	93.1 (86.7, 99.5)	
Saturated fat, en%	HPLG	68	11.9 (11.3, 12.6)	0.24
	MPMG	60	12.5 (11.8, 13.2)	
Cholesterol, mg/d	HPLG	68	351.8 (324.4, 379.2)	<0.001
	MPMG	60	224.6 (195.5, 253.7)	
MUFAs, g/d	HPLG	68	23.1 (21.1, 25.1)	0.96
	MPMG	60	23.0 (20.9, 25.2)	
PUFAs, g/d	HPLG	68	11.5 (10.7, 12.6)	0.11
	MPMG	60	10.3 (9.1, 11.4)	
LC n-3 PUFAs, mg/d	HPLG	68	641.1 (501.1, 781.0)	0.02
	MPMG	60	405.9 (256.9, 555.0)	
LA, g/d	HPLG	68	8.7 (7.7, 9.6)	0.52
	MPMG	60	8.3 (7.3, 9.2)	
ALA, g/d	HPLG	68	1.7 (1.4, 1.9)	0.03
	MPMG	60	1.2 (1.0, 1.5)	
Alcohol, en%	MPMG	60	3.4 (2.5, 4.3)	0.03
	HPLG	68	2.0 (1.0, 2.9)	

(Continued)

SUPPLEMENTAL TABLE 3 (Continued)

Measure	Group	n	6-mo[1]	Between Groups *P*-value[2]
Calcium, mg/d	HPLG	68	936.4 (859.5, 1013.3)	0.12
	MPMG	60	1026.0 (944.1, 1107.9)	
Iron, mg/d	HPLG	68	11.2 (10.5, 11.8)	0.68
	MPMG	60	10.9 (10.2, 11.7)	
Potassium, mg/d	HPLG	68	3425 (3244, 3605)	0.53
	MPMG	60	3341 (3148, 3533)	
Sodium, mg/d	HPLG	68	2154 (1961, 2347)	0.67
	MPMG	60	2092 (1887, 2298)	
Phosphorus, mg/d	HPLG	68	1622 (1542, 1702)	0.28
	MPMG	60	1557 (1472, 1642)	
Magnesium, mg/d	HPLG	68	426.0 (394.1, 457.8)	0.31
	MPMG	60	402.0 (368.1, 435.9)	
Zinc, mg/d	HPLG	68	11.7 (10.8, 12.6)	0.24
	MPMG	60	10.9 (9.9, 11.9)	
Selenium, μg/d	HPLG	68	108.3 (95.2, 121.5)	0.04
	MPMG	60	87.6 (73.6, 101.7)	
Iodine, μg/d	HPLG	68	164.1 (151.0, 177.2)	0.42
	MPMG	60	171.9 (158.1, 185.7)	
Thiamine, mg/d	HPLG	68	1.3 (1.2, 1.5)	0.30
	MPMG	60	1.5 (1.3, 1.6)	
Riboflavin, mg/d	HPLG	68	2.1 (1.9, 2.2)	0.92
	MPMG	60	2.0 (1.9, 2.2)	
Niacin (NE), mg/d	HPLG	68	25.8 (23.6, 28.1)	0.08
	MPMG	60	22.9 (20.5, 25.3)	
Vitamin B6, mg/d	HPLG	68	1.55 (1.4, 1.7)	0.25
	MPMG	60	1.4 (1.3, 1.6)	
DFE, μg/d	HPLG	68	356.7 (329.8, 383.6)	0.96
	MPMG	60	355.8 (327.2, 384.4)	
Vitamin B12, μg/d	HPLG	68	5.6 (5.1, 6.1)	0.01
	MPMG	60	4.6 (4.1, 5.2)	
Vitamin A (RE), μg/d	HPLG	68	1082 (952, 1212)	0.49
	MPMG	60	1016 (878, 1155)	
Vitamin C, mg/d	HPLG	68	126.4 (110.1, 142.7)	0.99
	MPMG	60	126.5 (109.2, 143.8)	
Vitamin E, mg/d	HPLG	68	11.5 (10.4, 12.6)	0.55
	MPMG	60	11.0 (9.8, 12.2)	

[1]Values are expressed as the adjusted mean (95% CI). GI, glycemic index; GL, glycemic load; HPLG, higher protein-lower glycemic index diet; MPMG, moderate protein-moderate glycemic index diet; NE, niacin equivalent; RE, retinol equivalent.

[2]*P*-value represent the difference in a variable among the two groups as assessed by 1-factor ANCOVA adjusted for each measurement 0-mo values, age and sex.
